# LncRNA *ELDR* promotes bladder cancer malignant progression by regulating the miR-1343-3p/*TRIM44* axis

**DOI:** 10.3389/fonc.2025.1685792

**Published:** 2025-11-21

**Authors:** Xiao Hu, Tianhang Xie, Xiao Xiao, Yuhua Mei

**Affiliations:** 1 Frontiers Science Center for Disease-related Molecular Network, Department of Orthopedic Surgery and Orthopedic Research Institute, West China Hospital, Sichuan University, Chengdu, Sichuan, China; 2 Department of Orthopedic Surgery and Orthopedic Research Institute, West China Hospital, Sichuan University, Chengdu, Sichuan, China; 3 Department of Urology, Chongqing University Fuling Hospital, Chongqing, China; 4 Department of Urology, The First Affiliated Hospital of Chongqing Medical University, Chongqing, China; 5 The First Affiliated Hospital of Chongqing Medical University, Chongqing, China

**Keywords:** bladder cancer, ELDR, ceRNA, miR-1343-3p, TRIM44

## Abstract

**Introduction:**

Bladder cancer (BCa) is one of the most prevalent genitourinary malignancies with high recurrence worldwide. A lack of reliable prognostic biomarkers and effective therapeutic targets hinders its treatment. Emerging evidence indicates that long noncoding RNAs (lncRNAs) are involved in human cancers, including BCa. While lncRNAs hold enormous promise, their specific roles and mechanisms in BCa remain largely unexplored. Here, we identify the lncRNA ELDR as a pivotal oncogenic driver in BCa.

**Methods:**

RT-qPCR was used to analyze the expression patterns of ELDR, miR-1343-3p and TRIM44. CCK-8, colony formation, EdU, and Transwell assays were used to detect the effect of ELDR on cell proliferation, migration, and invasion. The association between ELDR, miR-1343-3p and TRIM44 was analyzed by bioinformatics analysis and dual-luciferase reporter assay. Finally, the role of the ELDR-miR-1343-3p-TRIM44 axis in bladder cancer cell behavior was demonstrated.

**Results and Discussion:**

ELDR is significantly upregulated in BCa tissues, and its high expression correlates with aggressive clinicopathological features and predicts poor prognosis in BCa patients. Functional experiments demonstrate that ELDR enhances BCa cell proliferation, colony formation, migration, and invasion in vitro and accelerates tumor growth in vivo. Mechanistically, ELDR functions as a competitive endogenous RNA (ceRNA) by sequestering tumor-suppressive miR-1343-3p in the cytoplasm, which consequently leads to the upregulation of the oncogene TRIM44. Our findings unveil the ELDR/miR-1343-3p/TRIM44 axis as a crucial pathway in BCa progression, establishing ELDR as a promising prognostic biomarker and an attractive candidate for the development of targeted therapies.

## 1 Introduction

Bladder cancer, ranking as the second most prevalent malignancy of the genitourinary system, accounts for over 573,000 new cases globally each year (1). The characteristics of frequent recurrence and metastasis are the main reasons for its poor prognosis (2). The past decade has been important for strides in BCa detection and management. However, the prognosis for most patients remains poor. High recurrence rates and the aggressive course of disease mean an undesirable 5-year survival rate of BCa (3). Therefore, identifying novel genes and pathways involved in BCa is critical for developing new diagnostic and therapeutic approaches.

Long non-coding RNAs (lncRNAs) are defined as transcripts longer than 200 nucleotides with limited protein-coding potential. They have emerged as critical regulators of gene expression and cellular homeostasis (4). Although initially considered “transcriptional noise”, recent studies reveal that lncRNAs participate in diverse biological processes, including chromatin remodeling, RNA stability modulation, and signal transduction cascades. Interactions with proteins, DNA, or other RNAs enable these functions (5, 6). The roles of lncRNAs in disease pathogenesis—particularly cancer—are increasingly recognized. Numerous dysregulated lncRNAs operate as competitive endogenous RNAs (ceRNAs). They contribute to tumor initiation, metastasis, and therapy resistance by sponging tumor-suppressive miRNAs and derepressing oncogenic target genes (7, 8). For example, Xue et al. revealed that lncRNA *LUESCC* is highly expressed in ESCC and acts as a ceRNA to promote tumor proliferation, invasion, and migration by targeting the miR-6785-5p/*NRSN2* axis (9). Furthermore, Sheng et al. observed a clear upregulation of LINC01980 in HCC, which they correlated with a poor prognosis (10). Despite its association with various cancers, the role of lncRNA *ELDR* in BCa remains unclear. A previous study on oral cancer demonstrated that *ELDR* enhances tumor growth by promoting ILF3-cyclin E1 signaling (11). Additionally, *ELDR* is highly expressed and has potential as a biomarker of poor prognosis in the serum extracellular vesicles of breast cancer patients (12). These findings support the role of *ELDR* as an oncogenic molecule, suggesting the need for further studies on its mechanistic role in bladder carcinogenesis progression.

MicroRNAs (miRNAs) are small non-coding RNAs of 21–25 nucleotides. They are master regulators of carcinogenesis, modulating oncogenes and tumor suppressors at the post-transcriptional level (13). In BCa, dysregulated miRNAs drive malignant phenotypes—including proliferation, migration, invasion, and therapy resistance—through interactions with key genes (14, 15). For instance, Zhang et al. displayed that miR-15b-3p-mediated inhibition of ferroptosis could weaken bicalutamide sensitivity in prostate cancer (16). As a tumor suppressor gene, miR-1343-3p has been reported to be downregulated in expression in a variety of malignancies (17–19). Notably, Lai et al. revealed that miR-1343-3p can serve as an early screening marker for BCa (20). However, the potential mechanism of miR-1343-3p in BCa needs to be further explored.

Tripartite motif-containing 44 (TRIM44), a cytoplasmic and nuclear regulatory protein (21), is dysregulated in multiple human malignancies. Previous research indicated elevated TRIM44 levels in ovarian cancer drive tumor progression via activating the NF-kB pathway (22). It also correlates with aggressive clinical behavior in prostate cancer (23). A previous study demonstrated that TRIM44 was a risk factor affecting BCa (24); however, the underlying mechanism by which TRIM44 regulated BCa development remained unclear.

In this investigation, we demonstrate that highly expressed *ELDR* promotes malignant progression of BCa by targeting the miR-1343-3p/*TRIM44* axis, which is correlated with the poor prognosis. Therefore, *ELDR* may serve as a diagnostic biomarker and therapeutic target for BCa patients.

## 2 Materials and methods

### 2.1 Clinical tissue specimens collection

Primary BCa tumor tissues and paired adjacent normal tissues (at least 3 cm from the edge of cancer tissues) were acquired from 58 treatment-naïve patients undergoing curative resection at the First Affiliated Hospital of Chongqing Medical University (2019-2021). All cases received histopathological confirmation by two independent pathologists, with exclusion criteria encompassing any preoperative anticancer therapy. Immediately following surgical excision, tissues were snap-frozen in liquid nitrogen and cryopreserved at -80°C for molecular analyses. The study protocol obtained formal approval from the Medical Ethics Committee of the First Affiliated Hospital of Chongqing Medical University (2021-199), with written informed consent procured from all participants.

### 2.2 Cell culture and treatment

The human urothelial cell lines (SV-HUC-1, J82, T24, UM-UC-3, 5637, and RT4) were obtained from Wuhan Procell Biotechnology (Wuhan, China). Cells were maintained at 37°C in a humidified 5% CO_2_ incubator using the following media: SV-HUC-1 in F12K basal medium (Gibco, USA), J82 and UM-UC-3 in Dulbecco’s modified eagle media (DMEM, Gibco, USA), and T24/5637/RT4 in RPMI-1640 (Gibco, USA). All media contained 10% bovine serum (FBS; Procell, Wuhan, China) and 1% penicillin/streptomycin (Sangon, Shanghai, China).

### 2.3 Cell transfection, plasmids and oligonucleotides

Overexpression constructs for *ELDR* (pcDNA3.1-*ELDR*) and *TRIM44* (pcDNA3.1-TRIM44), along with *ELDR*-targeting shRNA (sh-*ELDR*) and miR-1343-3p mimics/inhibitors, were procured from Tsingke Biotechnology (Beijing, China). Corresponding negative control vectors and oligonucleotides were included. For gene modulation, cells were transfected using Lipofectamine™ 3000 (Invitrogen, USA) per the manufacturer’s protocol, with plasmid validation via Sanger sequencing.

### 2.4 RNA isolation, reverse transcription, and RT-qPCR

Total RNA isolation from cells and clinical specimens was performed with TRIzol reagent (Abclonal, China). The purified RNA underwent reverse transcription using the PrimeScript qRT-PCR kit (Abclonal, China). Subsequently, qRT-PCR analysis was conducted using the SYBR(R) Prime-Script RT-PCR kit (Abclonal, China) on an ABI 7500 Real-Time PCR Platform (Applied Biosystems, USA). Actin and U6 were used as internal controls, and gene expression levels were normalized to internal controls and quantified via the 2-ΔCt method. All samples were run in triplicate.

### 2.5 Cell counting kit-8 and colony formation assay

Cells were plated in 96-well plates at a density of 3 × 10³ cells per well. After incubation for 6 h, 24 h, 48 h, 72 h, and 96 h, the CCK-8 reagent (10 μL, Sangon) was incubated with each well for 1 h at 37°C, 5% CO_2_. Absorbance was measured at 450 nm using a microplate reader (Thermo Fisher, USA). About the colony formation assay, cells (1.5 × 10³/well) were seeded in 6-well plates and incubated at 37°C, 5% CO_2_ until colonies were visible. The cells were then fixed with 4% paraformaldehyde and stained with 0.1% crystal violet, and the colonies were counted.

### 2.6 Transwell migrating and invasion assay

For migration assays, cells were seeded in RPMI-1640 (500 μL) at 1×10^4^density in the upper chamber of Transwell inserts, with DMEM containing 10% FBS (1000 μL) in the lower compartment. Following 12 h incubation, migrated cells were fixed and stained with 0.1% crystal violet (30 min), then imaged using a microscope (Thermo Fisher, USA). Invasion assays followed identical procedures except for Matrigel coating (Corning; 1:8 dilution in serum-free medium) applied to the upper membrane prior to cell seeding.

### 2.7 Proliferation assay

Cell proliferation was assessed using an EdU Cell Proliferation Kit (C0071S, Beyotime), following the manufacturer’s protocol. Briefly, cells ^4^y of 4×10^4^ cells per well and pretreated for 24 hours. Subsequently, half of the medium was replaced with EdU-containing buffer (20 μM) for a 3-hour incubation period. Following fixation and permeabilization, the cells were incubated with click reaction solution and DAPI (1:1000 dilution). Three randomly selected fields per well were imaged, and the results were averaged for statistical analysis.

### 2.8 Western blot assay

Total protein was isolated from cells and tissues using RIPA lysis buffer (Beyotime) supplemented with phenylmethanesulfonyl fluoride (PMSF) at a 1:100 ratio. Following separation by SDS-PAGE, proteins were transferred onto PVDF membranes (EMD Millipore). The membranes were blocked for 1 hour in Tris-buffered saline (TBS) containing 5% skim milk and then incubated overnight at 4 °C with the following primary antibodies: PCNA (Proteintech, 10205-2-AP), GAPDH (Proteintech, 60004-1-Ig), TRIM44 (Abcam, ab236422), and β-actin (Proteintech, 66009-1-Ig). After washing, membranes were exposed to a species-matched secondary antibody for 1 hour at room temperature. Protein signals were finally detected using enhanced chemiluminescence (Cell Signaling Technology, USA).

### 2.9 Subcellular fractionation

Nuclear and cytoplasmic fractions were prepared from BCa cells cultured on 15 cm plates. Following two washes with ice-cold PBS, cells were gently scraped into a 15 mL Falcon tube. The resulting cell pellet was resuspended in 1 mL of hypotonic buffer (10 mM HEPES pH 8.0, 1.5 μM MgCl_2_, 10 mM KCl, 1 μM DTT) and incubated on ice for 15 minutes to induce cell swelling. After that, NP-40 was then added to the suspension to a final concentration of 1%, followed by brief vortexing (10 seconds) and centrifugation at 12,000 rpm for 2–3 minutes; the supernatant constituted the cytoplasmic fraction, while the pellet represented the nuclear fraction. Total RNA or protein from each compartment was subsequently extracted using Trizol (Abclonal, China) or RIPA lysis buffer (Beyotime), respectively, according to the manufacturers’ instructions. *ELDR* expression patterns across cellular fractions were assessed by RT-qPCR, using actin and U6 as internal controls for cytoplasmic and nuclear RNA, respectively. Finally, fraction purity was validated by immunoblotting with anti-GAPDH and anti-PCNA antibodies serving as cytoplasmic and nuclear markers, respectively.

### 2.10 Tumor xenograft *in vivo*

Six-week-old male BALB/c nude mice were randomly divided into three groups (n=5) and housed under SPF conditions. For xenograft experiments, T24 cells stably transfected with lentiviral vectors for shRNA against *ELDR* (1 × 10^6/100 μL) were injected subcutaneously into the back of nude mice. Tumor. Tumor size was assessed for three consecutive weeks, and after 21 days the mice received intraperitoneal pentobarbital (150 mg/kg). Death was confirmed by cervical dislocation, and the tumor tissue was weighed. The animal experiments were approved by the Ethics Committee of the First Affiliated Hospital of Chongqing Medical University.

### 2.11 Immunohistochemistry

Tumor specimens were fixed in 10% paraformaldehyde, decalcified with formic acid, and paraffin-embedded. Consecutive 4-μm sections were cut and subjected to deparaffinization and antigen retrieval (Dako, CA, USA). After blocking with goat serum, avidin, and biotin solutions, sections were incubated overnight with anti-Ki67 primary antibody (Abcam, 1:200, ab15580), followed by 1-hour incubation with secondary antibody (Abcam, 1:500, ab150077). Stained sections were finally examined under an inverted microscope (Nikon, Tokyo, Japan).

### 2.12 Dual luciferase reporter assay

The potential binding site between *ELDR* and miR-1343-3p was predicted using the starBase online database. A fragment of the human *ELDR* gene containing this site was amplified by PCR. Site-directed mutagenesis was performed on the predicted miR-1343-3p binding site within this fragment using a dedicated kit (Stratagene, USA). The resulting wild-type (wt) and mutant (mut) *ELDR* sequences were cloned into the PGL3 vector (GenePharma) to generate *ELDR*-wt-luc and *ELDR*-mut-luc reporter plasmids. Cells were then co-transfected with either *ELDR*-wt-luc or *ELDR*-mut-luc plasmids together with miR-1343-3p mimics or negative control mimics (NC), using Lipofectamine™ 3000 (Invitrogen). Luciferase activity was measured 48 hours post-transfection using the Dual-Luciferase^®^ Reporter Assay System (Promega, WI, USA), following the manufacturer’s instructions. This identical experimental approach was applied to confirm the interaction between miR-1343-3p and *TRIM44*.

### 2.13 AGO2-RIP and MS2-RIP

The Magna RIP Kit (Millipore, USA) was used for RNA immunoprecipitation (RIP) experiments in accordance with product guidelines. 5 µg of control IgG or anti-Argonaute 2 (AGO2) antibody (Abcam, USA) was added to pre-cleared BCa cell lysates in RIP lysis solution, which were then rotated and incubated for an entire night at 4 °C. After adding protein A/G magnetic beads to separate RNA-protein complexes, bound RNAs were released by proteinase K digestion. Using quantitative real-time PCR, the quantities of precipitated target RNA were measured.

For MS2-RIP validation of endogenous lncRNA-*ELDR*/miR-1343-3p binding, wild-type lncRNA-*ELDR* and its miR-1343-3p binding-site mutant were cloned into pcDNA3.1-MS2 (12×) to create pcDNA3.1-MS2-*ELDR*-WT and pcDNA3.1-MS2-*ELDR*-MUT constructs for MS2-RIP validation of endogenous lncRNA-*ELDR*/miR-1343-3p binding. In addition to pcDNA3.1-MS2/GFP (expressing MS2-GFP fusion protein), BCa cells were co-transfected with pcDNA3.1-MS2 (vector control), pcDNA3.1-MS2-*ELDR*-WT, or pcDNA3.1-MS2-*ELDR*-MUT. Using the previously indicated RIP technique, RNA complexes were immunoprecipitated with anti-GFP antibody (Abcam, USA) during a 48-hour incubation period.

### 2.14 Statistical analysis

Data from at least three independent experiments are expressed as mean ± SD and analyzed using GraphPad Prism 9.5.1. Group comparisons employed Student’s t-test (two groups) or one-way ANOVA (multiple groups). Associations between *ELDR* expression and clinicopathological features were assessed by the chi-square test. Survival distributions were analyzed with Kaplan-Meier curves and log-rank testing. Correlation analyses used Pearson’s coefficients. All tests were two-tailed, with *p* < 0.05 considered statistically significant.

## 3 Results

### 3.1 *ELDR* is significantly upregulated in BCa tissues and cell lines, correlating with poor prognosis

**Figure 1:**
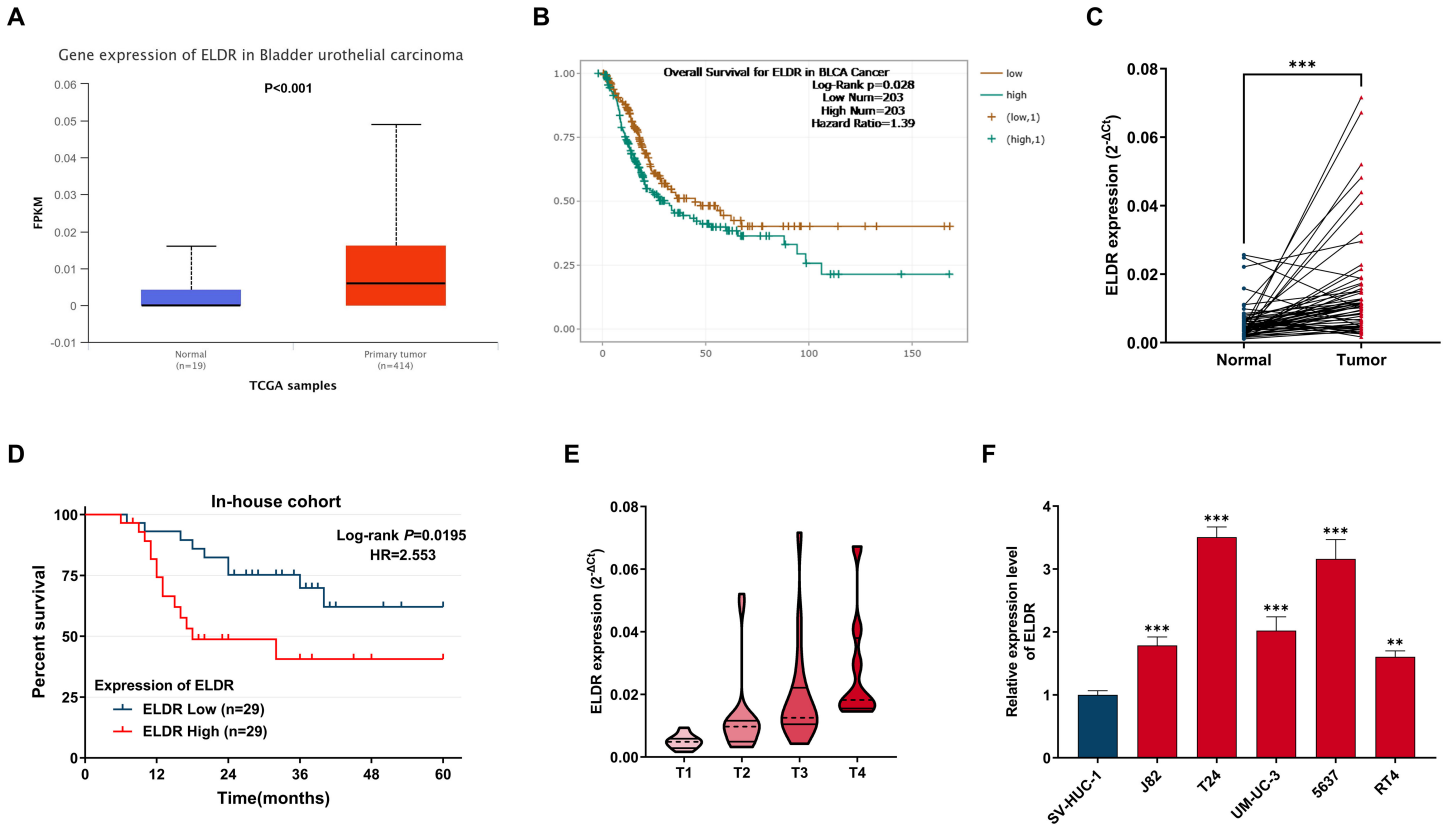
LncRNA *ELDR* expression is over-presented in BCa tumors tissues and cells, and predicts poor prognosis in BCa patients. **(A)***ELDR* expression in tumor and normal tissues in UALCAN database. **(B)** The overall Survival for *ELDR* in BCa from starBase database. **(C)** The expression of *ELDR* in an in-house cohort of 58 paired BCa tumor and adjacent normal tissues is shown. **(D)** The correlation between the expression of *ELDR* and the prognosis of patients for the BCa in in-house cohort. **(E)***ELDR* expression in BCa tissue of TNM stage 1-2 (n=33) and BCa tissues of TNM stage 3-4 (n=25) were determined using qRT-PCR assay. **(F)***ELDR* expression in SV-HUC-1, J82, T24, UM-UC-3, 5637, RT4 cells were detected by qRT-PCR assay. Data were shown as mean ± SD of three. **P <0.01, ***P < 0.001.

To explore the correlation of *ELDR* with BCa development, we analyzed its expression in TCGA-BCa data via the UALCAN web portal. As shown in **Figure 1A**, *ELDR* expression was substantially upregulated in BCa tissues compared to normal tissues. Subsequently, using the starBase database, we found that high *ELDR* levels were markedly associated with poor prognosis in BCa patients (**Figure 1B**). To validate these findings and further strengthen the clinical significance of *ELDR*, we obtained tumor tissues and paired adjacent normal tissues from 58 bladder cancer patients. The result of the qRT-PCR assay suggested that the *ELDR* expression was obviously elevated in BCa tissues (**Figure 1C**, **Supplementary Table S1**). Moreover, we found that higher *ELDR* levels predicted a worse prognosis and enhanced malignant progression (**Figures 1D, E**, **Supplementary Table S1**). Multivariate analysis of clinicopathological characteristics identified that high levels of *ELDR* were independently associated with tumor size, tumor invasion depth, and TNM stage within our cohort (**Supplementary Table S2**). Consistently, qRT-PCR analysis confirmed that *ELDR* levels were markedly higher in BCa cell lines (J82, T24, UM-UC-3, 5637, and RT4) than in the normal uroepithelial sv-HUC-1 line (**Figure 1F**). In summary, these results suggested that *ELDR* was pronouncedly elevated in BCa tissues and cell lines, correlating with clinicopathological features.

### 3.2 *ELDR* promotes BCa cells malignant behaviors *in vitro*

**Figure 2:**
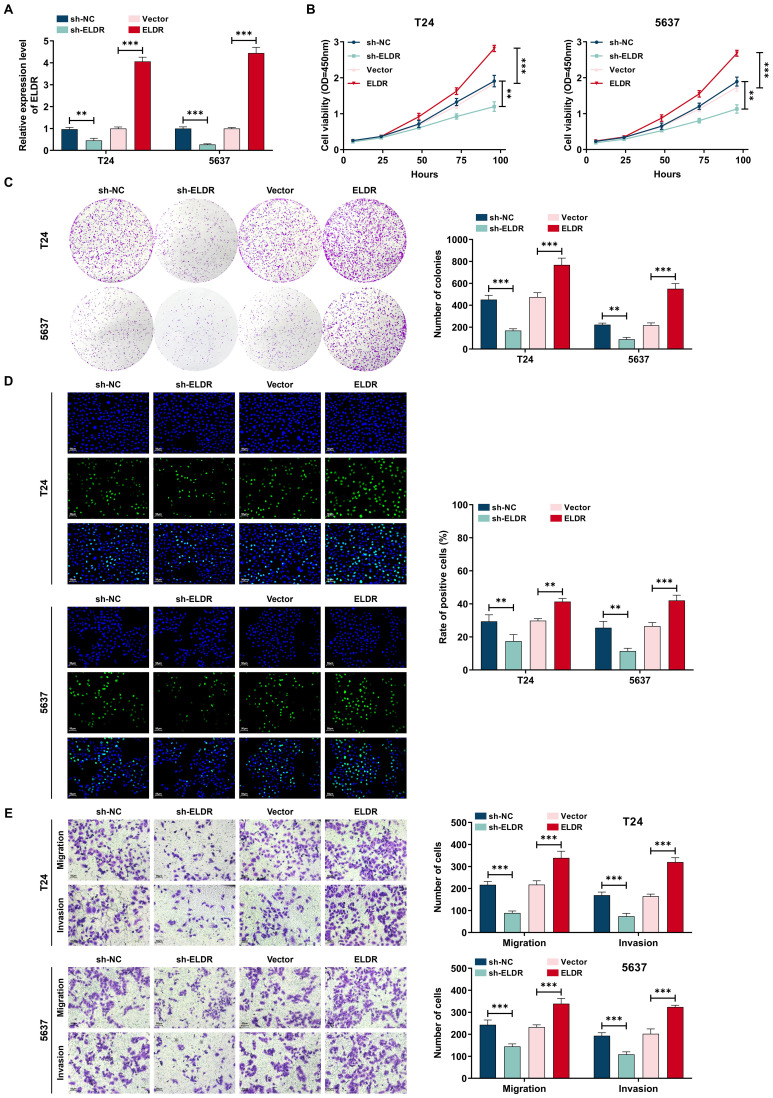
*ELDR* promotes cell proliferation, colony formation, migration, and invasion in BCa cells. T24 and 5637 were transfected with sh-NC, sh-*ELDR*, pcDNA3.1 or pc-*ELDR*. **(A)***ELDR* expression was assessed using qRT-PCR assay. **(B-D)** CCK-8, colony formation, and EDU assays were employed to determine cell proliferation. **(E)** Cell migration and invasion were detected using transwell assay. Data were shown as mean ± SD. **P <0.01, ***P < 0.001.

To investigate the potential biological functions of *ELDR* in BCa progression, we performed gain- and loss-of-function experiments. Transfection with pcDNA3.1-*ELDR* significantly increased *ELDR* levels in both T24 and 5637 cells, while sh-*ELDR* transfection effectively reduced its expression (**Figure 2A**). Subsequently, we performed CCK-8, colony formation, transwells and EdU assays *in vitro*. As shown in **Figures 2B-E**, *ELDR* knockdown substantially inhibited proliferation, colony formation, migration, and invasion in both cell lines, whereas its overexpression potentiated these malignant phenotypes. Taken together, *ELDR* promotes the malignant behaviors of BCa cells.

### 3.3 *ELDR* knockdown suppresses tumor growth *in vivo*

**Figure 3:**
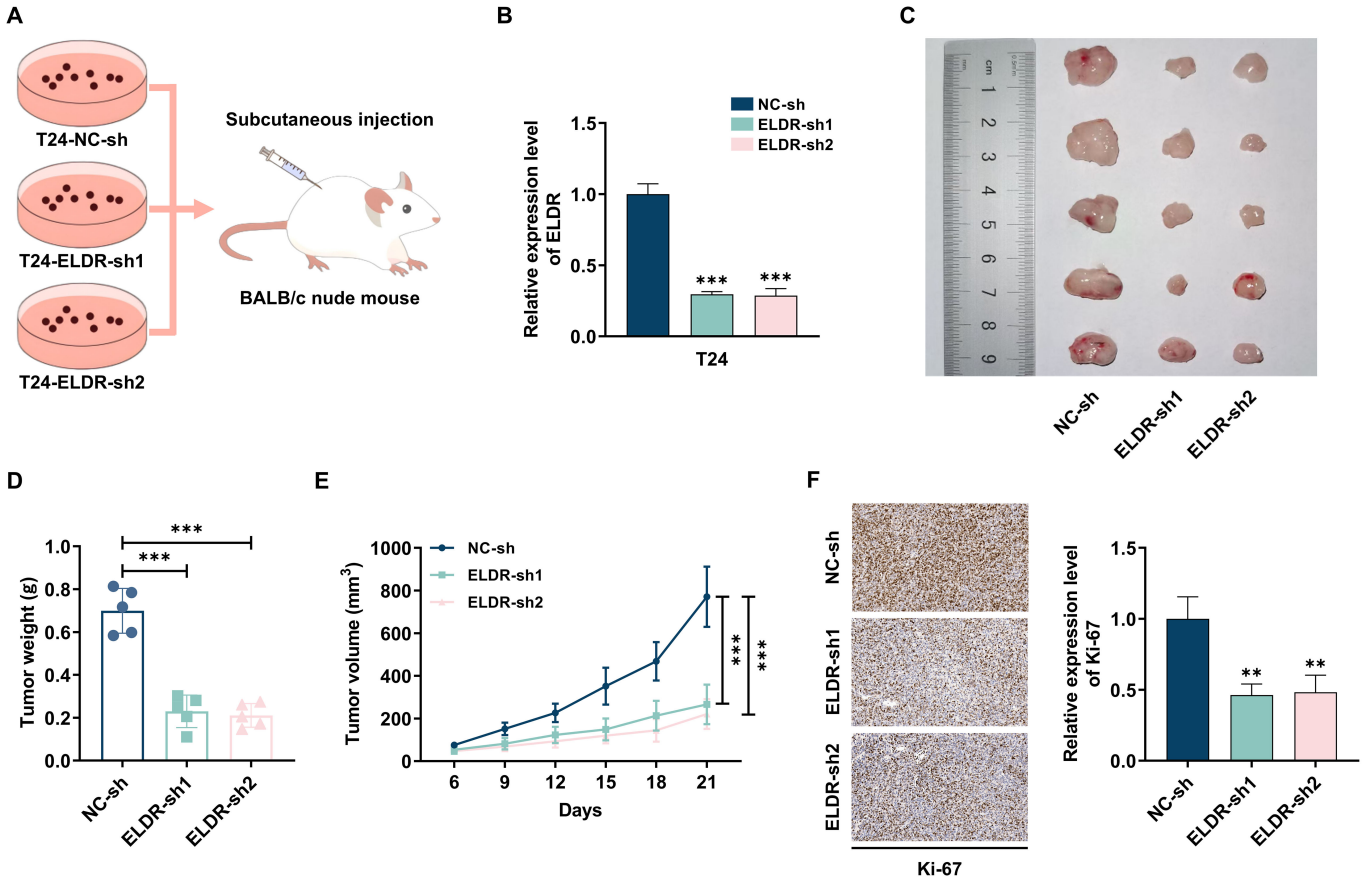
*ELDR* knockdown inhibited tumor formation *in vivo*. **(A, B)** T24 cells transfected with sh-*ELDR* and sh-NC were injected subcutaneously into the back of nude mice. **(C-E)** The tumors were collected, and the size and weight of tumors were measured. **(F)** The level of Ki67 in tumor tissues was evaluated using IHC. Data were shown as mean ± SD. **P <0.01, ***P < 0.001.

Next, to further assess the effects of *ELDR* on BCa tumorigenesis *in vivo*, we established subcutaneous xenograft models by injecting nude mice with T24 cells stably expressing control shRNA or two independent shRNAs targeting *ELDR* (**FigureS 3A, B**). Tumors derived from sh-*ELDR*-infected T24 cells exhibited significantly reduced growth rates and smaller volumes compared to those from control shRNA-infected cells (**Figures 3C-E**). Additionally, immunohistochemical (IHC) analysis revealed a striking reduction in the expression of the proliferation marker Ki67 in tumors from the sh-*ELDR* group compared to the control group (**Figure 3F**). Collectively, these results demonstrate that *ELDR* inhibition could suppress BCa tumor growth *in vivo*.

### 3.4 *ELDR* localizes to the cytoplasm and functions as a miR-1343-3p sponge

**Figure 4:**
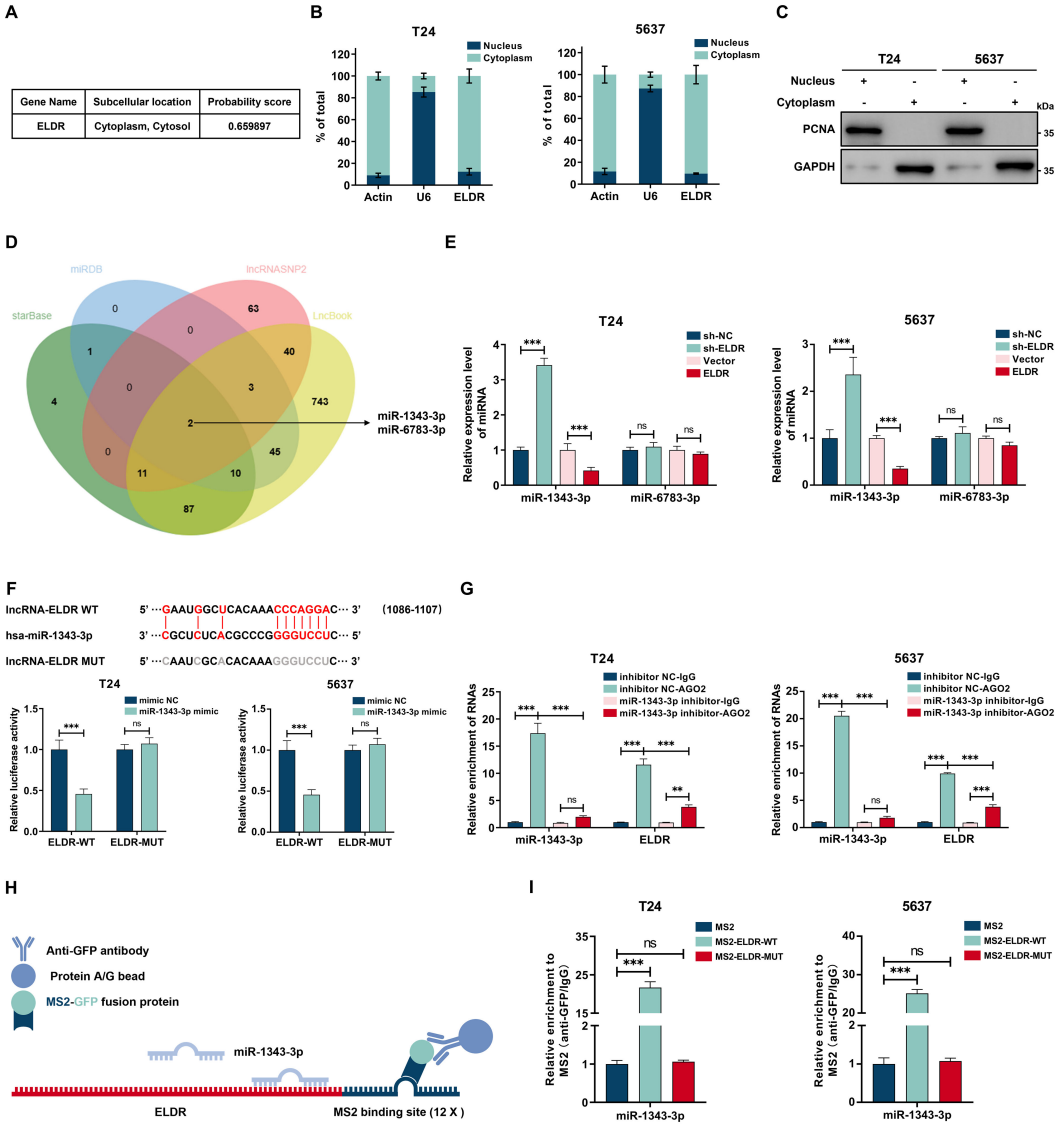
Cytoplasmic *ELDR* acted as a sponge of miR-1343-3p. **(A)** Subcellular localization of *ELDR* in cells predicted by iLoc-lncRNA databese. **(B)** The subcellular localization of *ELDR* in T24 (left panel) and 5637 (right panel) cells was determined by nuclear and cytoplasmic fractionation experiment followed by RT-qPCR analysis. **(C)** The nuclear and cytoplasmic fractionations as described in B were subjected to immunoblotting analysis. **(D)** Online predicting the *ELDR* target miRNAs. Venn gram showed the intersection miRNAs. **(E)** qRT-PCR assays were performed to screen target miRNAs of *ELDR* by silencing or overexpressing *ELDR* in BCa cell lines. **(F)** Online predicting of the interaction sites between *ELDR* and miR-1343-3p, T24 and 5637 cells were transfected with reporters containing wild-type or mutated *ELDR* in the presence or absence of negative control miRNA mimic or miR-1343-3p mimic followed by luciferase activity measurement. **(G)** RIP assays were used to pulldown the endogenous RNA associated with AGO2 in T24 and 5637 cells transfected with miR-1343-3p inhibitor or inhibitor NC, and the relative levels of *ELDR* and miR-1343-3p were measured and normalized according to the result in IgG group. **(H)** Schematic delineating the strategy of MS2-RIP. **(I)** MS2-RIP assays in T24 and 5637 cells followed by qRT-PCR examining the endogenous binding between *ELDR* and miR-1343-3p. Data were shown as mean ± SD, **P <0.01, ***P < 0.001.

We explored the molecular mechanisms of *ELDR*-mediated BCa tumorigenesis. Considering that the mechanisms and functions are dictated by subcellular localization, we first defined the compartmental distribution of ELDR (25). Bioinformatics prediction using the iLoc-lncRNA database indicated its predominant cytoplasmic accumulation (**Figure 4A**). This distribution was confirmed experimentally through subcellular fractionation and qRT-PCR in both T24 and 5637 cells, with fraction purity verified by immunoblotting for GAPDH (cytoplasmic marker) and PCNA (nuclear marker) (**Figures 4B, C**). Based on the established role of cytoplasmic noncoding RNAs in miRNA sequestration and target derepression, we hypothesized that *ELDR* acts as a molecular sponge for specific miRNAs to regulate downstream oncogenic genes (26).

To identify the underlying mechanism, potential miRNAs binding to *ELDR* were predicted using the starBase, miRDB, lncRNASNP2, and LncBook databases. By intersecting the four databases, we obtained two predicted miRNAs, miR-1343-3p and miR-6783-3p (**Figure 4D**). We performed qRT-PCR assays to confirm that miR-1343-3p is the direct downstream target of *ELDR*, as *ELDR* overexpression decreased miR-1343-3p levels while its knockdown upregulated them in both cell lines (**Figure 4E**). To ascertain the interaction between *ELDR* and miR-1343-3p, we predicted their binding sites using starBase and constructed the wild-type (WT) and mutant (MUT) *ELDR* luciferase reporter genes respectively. We performed the dual-luciferase assays, and the results of dual-luciferase indicated that the luciferase activity of *ELDR*-WT, but not MUT, was markedly attenuated by miR-1343-3p (**Figure 4F**, **Supplementary Figure S1A**). Furthermore, *ELDR* expression levels were significantly higher than those of miR-1343-3p in both cell lines, supporting its efficacy as a competing endogenous RNA in binding miR-1343-3p (**Supplementary Figure S1B**). It is well established that lncRNA-miRNA interactions depend on the AGO2 complex; therefore, we performed AGO2-RIP assays in both cell lines, which revealed that *ELDR* directly bound to the AGO2-containing miR-1343-3p ribonucleoprotein complex, but this interaction was obviously reduced upon inhibition of miR-1343-3p (**Figure 4G**, **Supplementary Figure S1C**). Subsequently, MS2-RIP assays demonstrated that endogenous miR-1343-3p was enriched by the MS2-*ELDR*-WT complex, further validating endogenous binding between *ELDR* and miR-1343-3p (**Figures 4H, I**). Besides, in BCa tissues from our in-house cohort, the miR-1343-3p expression level was significantly downregulated compared to paired adjacent normal tissues, and correlation analyses revealed that the *ELDR* expression level was negatively correlated with miR-1343-3p (**Supplementary Figures S1D, E**, **Supplementary Table S1**). Altogether, *ELDR* localized in the cytoplasm sponges miR-1343-3p to suppress its expression in BCa.

### 3.5 MiR-1343-3p inhibitor rescued malignant phenotypes suppressed by *ELDR* knockdown in BCa cells

**Figure 5:**
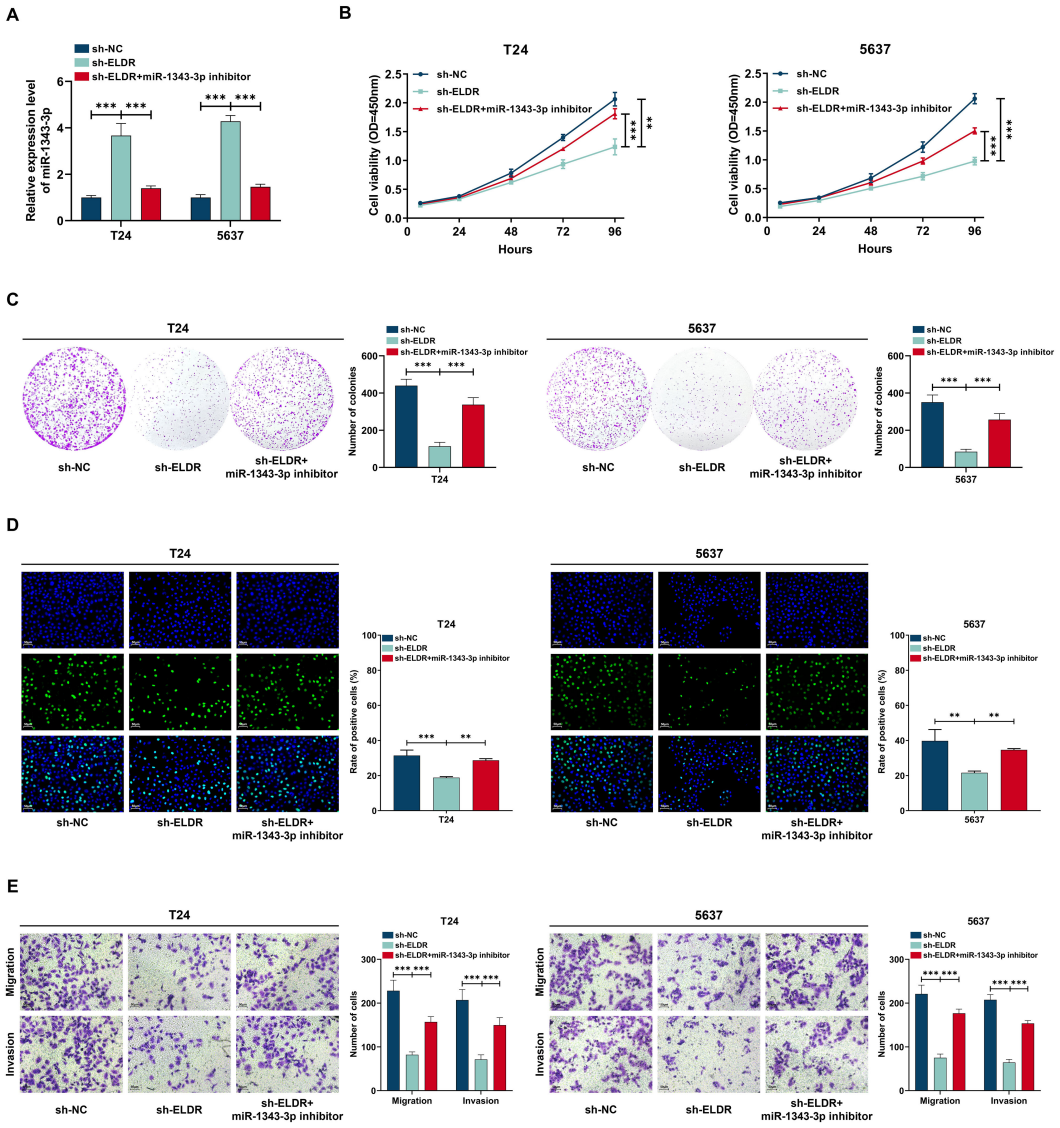
MiR-1343-3p inhibitor reversed the inhibitory effect of *ELDR* knockdown on the malignant phenotypes of BCa cells. T24 and 5637 cells were classified into: sh-NC group, sh-*ELDR* group, sh-*ELDR*+miR-1343-3p inhibitor group. **(A)** MiR-1343-3p was determined using qRT-PCR assay. **(B-D)** Cell proliferation was determined using CCK-8 assay, colony formation, and EDU assay. **(E)** Cell migration and invasion were determined using transwell assay. Data were expressed as mean ± SD, **P <0.01, ***P < 0.001.

To explore the functional significance of the *ELDR*/miR-1343-3p axis in BCa progression, we allocated T24 and 5637 cells into three experimental groups: shRNA negative control (sh-NC), *ELDR*-knockdown (sh-*ELDR*), and combined *ELDR*-knockdown with miR-1343-3p inhibitor (sh-*ELDR* + miR-1343-3p inhibitor), followed by CCK-8, colony formation, EdU and transwell assays (**Figures 5A**). As displayed in **Figures 5B-E**, *ELDR* knockdown markedly suppressed proliferation, colony formation, migration, and invasion capacities in both cell lines. Conversely, the miR-1343-3p inhibitor attenuated these suppressive effects, rescuing malignant phenotypes. Collectively, *ELDR* functions as a driver in BCa progression by targeting miR-1343-3p.

### 3.6 *ELDR* functions as a ceRNA for miR-1343-3p to upregulate TRIM44 expression

**Figure 6:**
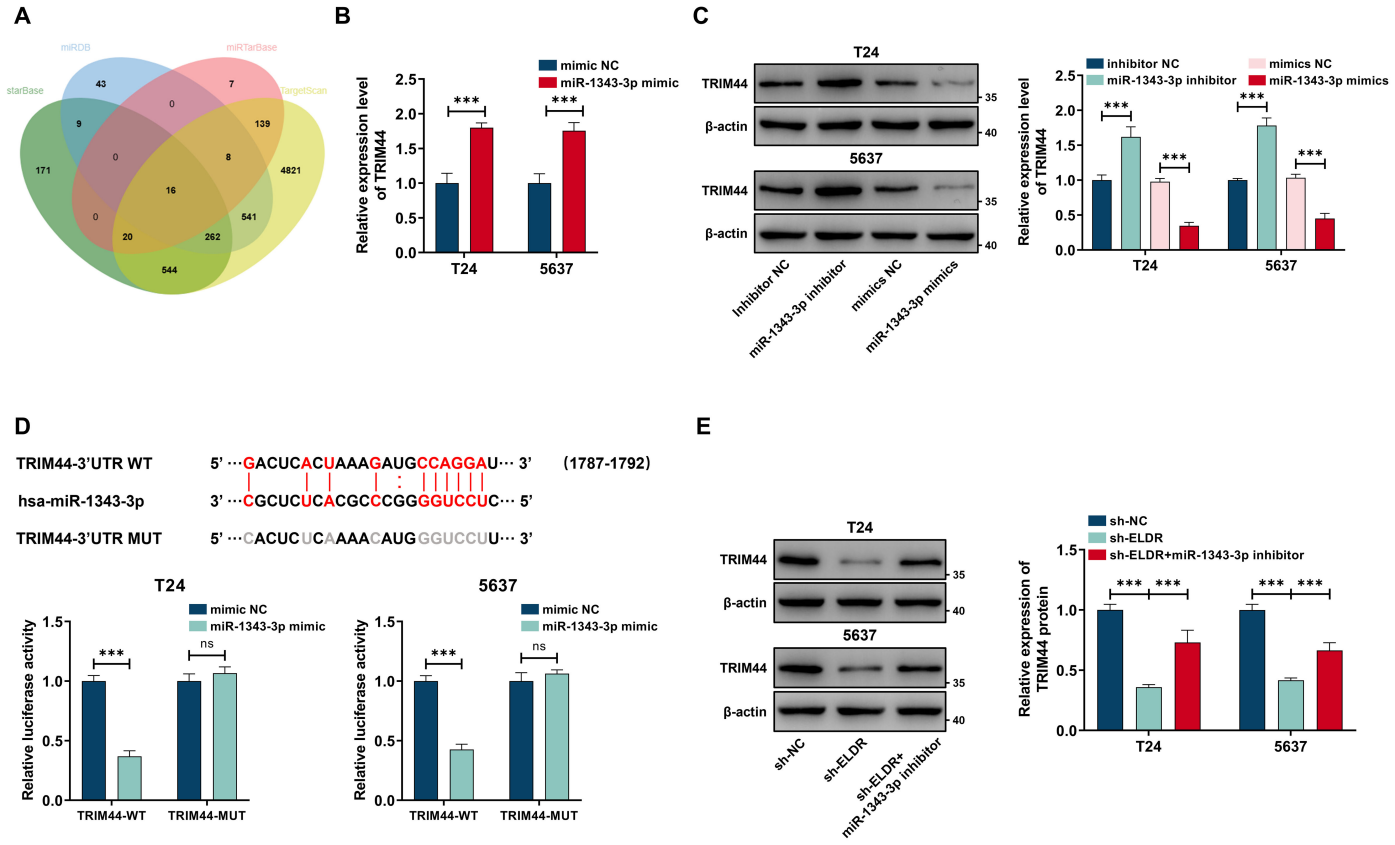
*ELDR* positively regulated TRIM44 expression through sponging miR-1343-3p. **(A)** Online predicting the miR-1343-3p target genes. Venn gram showed the intersection genes. **(B)** TRIM44 mRNA expression in T24 and 5637 cells after miR-1343-3p overexpression was determined using qRT-PCR. **(C)** TRIM44 proteins levels after transfection of miR-1343-3p mimic and inhibitor in the above two cell lines. **(D)** Online predicting of the interaction sites between miR-1343-3p and TRIM44, and luciferase reporter assays demonstrated TRIM44 was a direct target of miR-1343-3p. **(E)** TRIM44 expression in 5637 and T24 cells after sh-*ELDR* transfection or sh-*ELDR* and miR-1343-3p inhibitor co-transfection was detected by western blot. Data were expressed as mean ± SD, **P <0.01, ***P < 0.001.

Given that miRNAs exert their function via regulating the expression of target genes, we employed four bioinformatic databases—starBase, miRDB, miRTarBase, and TargetScan—to predict candidate targets of miR-1343-3p. As shown in **Figure 6A**, sixteen targets overlapped in the prediction results of these four databases. We performed qRT-PCR assays, and the results of showed that only the expression level of *TRIM44* mRNA was sharply decreased after treatment with miR-1343-3p mimic in T24 and 5637 cells (**Supplementary Figure S1F**). Conversely, the miR-1343-3p inhibitor promoted the *TRIM44* mRNA expression level in both cell lines (**Figure 6B**). A similar pattern of expression was observed for the TRIM44 protein by western blot assay (**Figure 6C**). Subsequently, we confirmed that miR-1343-3p directly bound to the 3’-UTR of *TRIM44* via a dual-luciferase reporter assay. Transfection with the miR-1343-3p mimic significantly suppressed luciferase activity driven by the wild-type (WT) *TRIM44* 3’-UTR reporters relative to the miR-NC control in both cell lines, whereas activity of the mutant (MUT) reporter was unaffected (**Figure 6D**). We also detected TRIM44 expression level in our in-house cohort using western blot and qRT-PCR assays. The result showed that TRIM44 was markedly upregulated in the tumor tissues compared with paired adjacent normal tissues (**Supplementary Figures S1G, SH**). Moreover, *TRIM44* mRNA expression level showed a positive relationship with *ELDR* and an inverse association with miR-1343-3p (**Supplementary Figures SI, SJ**, **Supplementary Table S1**). Finally, silencing *ELDR* expression substantially diminished TRIM44 protein levels in both cell lines, whereas this reduction was reversed by the miR-1343-3p inhibitor (**Figure 6E**). In summary, *ELDR* promotes TRIM44 expression via sponging miR-1343-3p.

### 3.7 TRIM44 overexpression rescued the inhibition of *ELDR* knockdown on the malignant phenotypes of BCa cells

**Figure 7:**
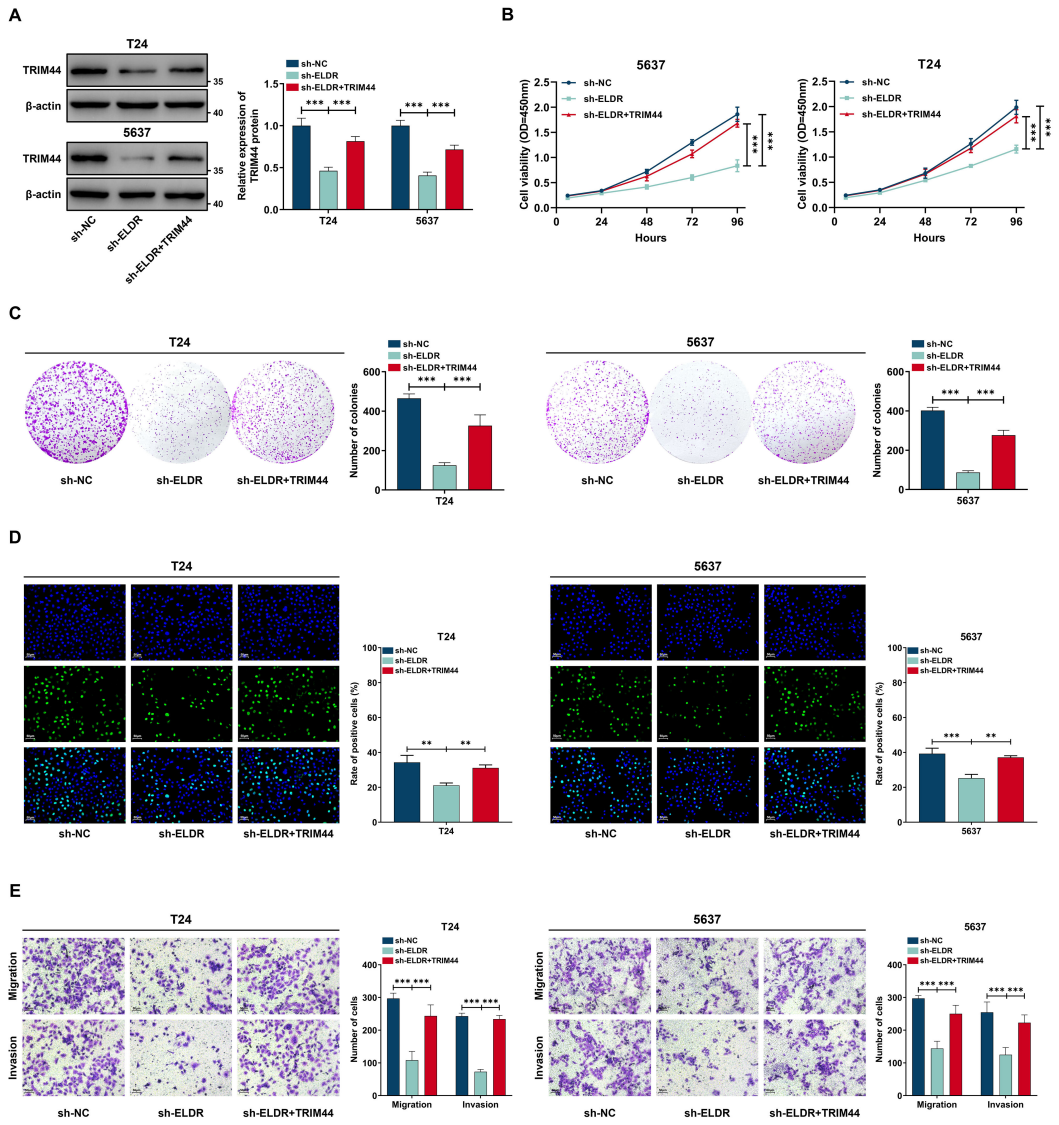
TRIM44 overexpression reversed the inhibitory effect of *ELDR* knockdown on the malignant phenotypes of BCa cells. T24 and 5637 cells were classified into: sh-NC group, sh-*ELDR* group, and sh-*ELDR*+TRIM44 group. **(A)** Western blot was employed to asses TRIM44 level in T24 and 5637 cells. **(B-D)** CCK-8 assay, colony information assay, and EDU assay were employed to determine cell proliferation. **(E)** Cell migration and invasion were determined using transwell assay. Data were expressed as mean ± SD, **P <0.01, ***P < 0.001.

To determine whether *TRIM44* mediates *ELDR* oncogenic function in BCa, we performed rescue experiments via restoring TRIM44 expression. *ELDR* knockdown significantly reduced TRIM44 protein levels in T24 and 5637 cells, an effect rescued by pcDNA3.1-*TRIM44* transfection (**Figure 7A**). Subsequent functional assays revealed that silencing *ELDR* inhibited BCa cell proliferation, colony formation, migration, and invasion in both cell lines (**Figures 7B-E**). Notably, TRIM44 restoration abolished *ELDR*-silencing-induced suppression of these malignant phenotypes (**Figures 7B-E**). Collectively, TRIM44 overexpression rescues the inhibitory effects of *ELDR* depletion on BCa oncogenicity.

## 4 Discussion

As the primary genitourinary malignancy, the prognosis of BCa remains poor due to a lack of effective diagnostic biomarkers and therapeutic drugs (27). It has been reported that abnormal proliferation is the main reason for the occurrence and malignant progression of BCa (28). Therefore, there is an urgent need to better understand the underlying molecular mechanisms of BCa progression and improve the survival of BCa patients. LncRNAs have received increasing attention due to their important roles in the development and progression of human cancers, including BCa (29–32). For example, *TUG1* (33) and *SNHG16* (34) showed overexpression in BCa tumor tissues and served as bad predictors of overall survival. However, the exact role of lncRNAs in BCa remains poorly understood. A comprehensive understanding of the mechanisms of lncRNAs will help reveal promising biomarkers and therapeutic targets for BCa patients. While the ceRNA paradigm and the oncogenic role of TRIM44 have been documented in various cancer types, the upstream regulators that dictate TRIM44 expression in BCa remain poorly characterized.

In this study, we focused our attention on a lncRNA *ELDR* that has not been reported in BCa and whose function is still unclear. Our work provides the first evidence that *ELDR* functions as a key oncogenic lncRNA in BCa by operating as a competitive endogenous RNA (ceRNA). We found that the expression level of *ELDR* was obviously upregulated in BCa tissues compared to the paired adjacent normal tissues, correlating with tumor size, invasion depth, TNM stage and poor prognosis in BCa patients. Functional experiments displayed that silencing *ELDR* inhibited the BCa cell proliferation, colony formation, migration, and invasion, whereas overexpression of *ELDR* showed the opposite effect. The tumor-promoting effect of *ELDR in vivo* was further validated. While other well-characterized oncogenic lncRNAs in BCa, such as *UCA1* and *MALAT1*, have been associated with specific processes like chemoresistance and metastasis, respectively, our date defines a distinct role for *ELDR*. We demonstrate that *ELDR* directly sequesters miR-1343-3p, which in turn leads to the derepression of its target oncogene, *TRIM44*. These results motivate us to further explore the biological mechanism of *ELDR* in BCa.

TRIM44, a member of the tripartite motif (TRIM) family E3 ubiquitin ligases, is recognized for its involvement in protein stability regulation and signal transduction (35, 36). Accumulating evidence indicates that the crucial roles of TRIM44 in the development of various types of malignant tumors. TRIM44 overexpression was confirmed to be associated with the malignant phenotype in gastric cancer (37), lung adenocarcinoma (38), and ovarian cancer (22). The precise molecular effects triggered by the upregulation of TRIM44 in bladder cancer require further investigation. our study provides the first evidence of TRIM44 dysregulation in BCa. Moreover, TRIM44 expression was positively correlated with that of ELDR. Importantly, the inhibitory effect of ELDR silencing on the BCa malignant phenotype were significantly attenuated in the presence of TRIM44 overexpression. Based on these results, we concluded that ELDR promotes the progression of BCa, at least to some extent, by stimulating the expression of TRIM44. There is already a lot of proof that TRIM44 makes the PI3K protein more stable and speeds up the PI3K/AKT signaling pathways in different malignancies. This suggests that TRIM44 overexpression caused by *ELDR* probably acts in bladder cancer by making this essential cancer-causing pathway work too hard. This potential association provides a plausible explanation for the enhanced growth capacity observed in *ELDR*-overexpressing cells. Concurrently, the potent pro-migration and invasion phenotypes induced by *ELDR* strongly suggest its involvement in regulating epithelial-mesenchymal transition (EMT)—a core mechanism of cancer metastasis. Future research focusing on this pathway’s effects on key EMT transcription factors and biomarkers (such as E-cadherin, N-cadherin, and vimentin) will be crucial for comprehensively elucidating its role in bladder cancer dissemination.

Our findings indicate promising potential for translational applications. From a diagnostic perspective, the substantial increase of *ELDR* in BCa tissues and its association with aggressive disease highlight its potential as a predictive biomarker. Identifying *ELDR* levels in liquid biopsies, such as serum or urine extracellular vesicles, may aid in the creation of non-invasive diagnostics for early detection and risk assessment. From a therapeutic standpoint, the unique and heightened expression of *ELDR* in malignancies renders it a compelling target for targeted therapies. Targeting lncRNAs poses technical challenges; however, innovative methodologies, including the application of antisense oligonucleotides (ASOs) or small interfering RNAs (siRNAs) specifically engineered to inhibit *ELDR*, may provide a novel and precise therapeutic approach to disrupt this carcinogenic pathway.

It must be acknowledged that this study has limitations. First, our clinical relevance analysis is based solely on a single-center cohort with limited sample size. To establish the post-rain value of *ELDR*, validation through independent, multicenter, large-scale prospective cohorts is essential. Second, although *in vitro* and *in vivo* data strongly support the functional role of the *ELDR*/miR-1343-3p/*TRIM44* axis, the direct clinical utility of *ELDR* and its feasibility for patient treatment remain to be verified. Finally, confirmatory validation of the proposed downstream pathways—particularly the direct activation mechanism of the PI3K/AKT pathway and the role of TRIM44 in inducing epithelial-mesenchymal transition (EMT) in bladder cancer—has become the core focus of our current research.

**Figure 8:**
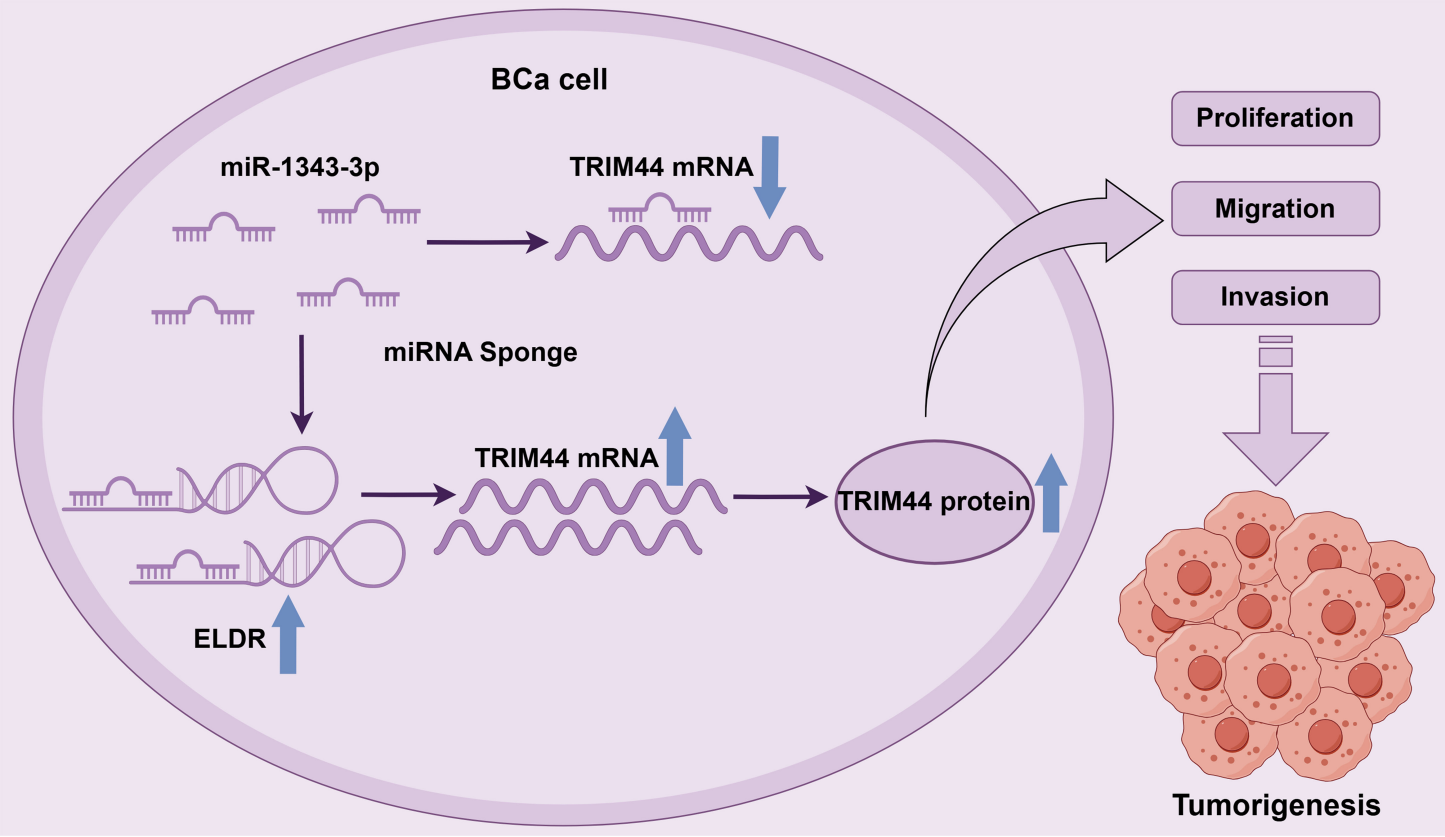
The schematic diagram of LncRNA ELDR promotes bladder cancer malignant progression by regulating the miR-1343-3p/TRIM44 axis.

Collectively, these findings delineate the *ELDR*/miR-1343-3p/*TRIM44* axis as a functionally independent pathway that expands the known regulatory network of lncRNAs in bladder cancer pathogenesis (**Figure 8**).

## Data Availability

The original contributions presented in the study are included in the article/**Supplementary Material**. Further inquiries can be directed to the corresponding authors.
