# Supplementary material for: LncRNA ELDR promotes bladder cancer malignant progression by regulating the miR-1343-3p/TRIM44 axis
Source: Front Oncol. 2025 Nov 21;15:1685792. doi: 10.3389/fonc.2025.1685792 (PMC12678138; doi:10.3389/fonc.2025.1685792)
Supplement: Supplementary file 1 [file DataSheet1.docx]

Supplementary Material

# Supplementary Figure S1.



(A) The relative levels of miRNAs in T24 and 5637 cells transfected with miRNA mimics. (B) The expression levels of miR-1343-3p and ELDR in T24 and 5637 cells. (C) The relative levels of miRNAs in T24 and 5637 cells transfected with miRNA inhibitor. (D) The expression of miR-1343-3p in an in-house cohort of 58 paired BCa tumor and adjacent normal tissues is shown. (E) Correlation analysis of ELDR expression and miR-1343-3p expression in BCa tissues. (F) qRT-PCR assays were conducted to screen target genes of miR1343-3p by overexpressing miR-1343-3p in BCa cells. (G, H) Western blot and qRT-PCR was employed to asses TRIM44 expression level in BCa tumor tissues and paired adjacent normal tissues. (I) Correlation analysis of ELDR expression and TRIM44 expression in BCa tissues. (J) Correlation analysis of miR-1343-3p expression and TRIM44 expression in BCa tissues.

# Original Western Blot Figures

**Figure 6C**

**T24**

**TRIM44**

**
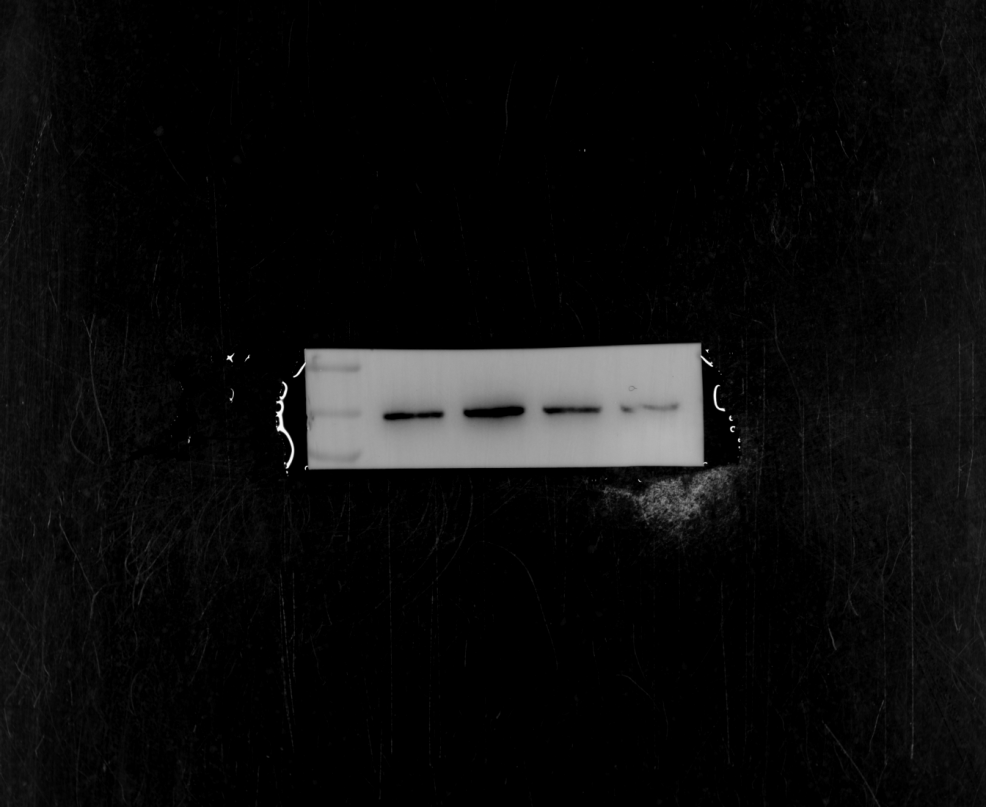
**

**β-actin**

**
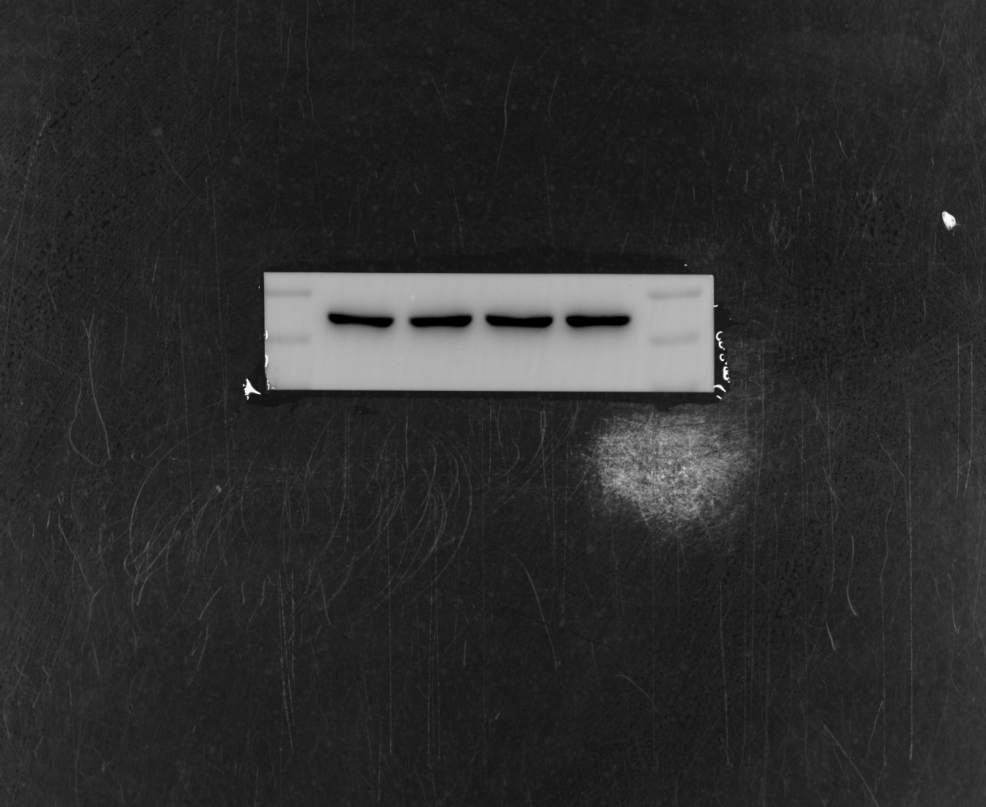
**

**5637**

**TRIM44**

**
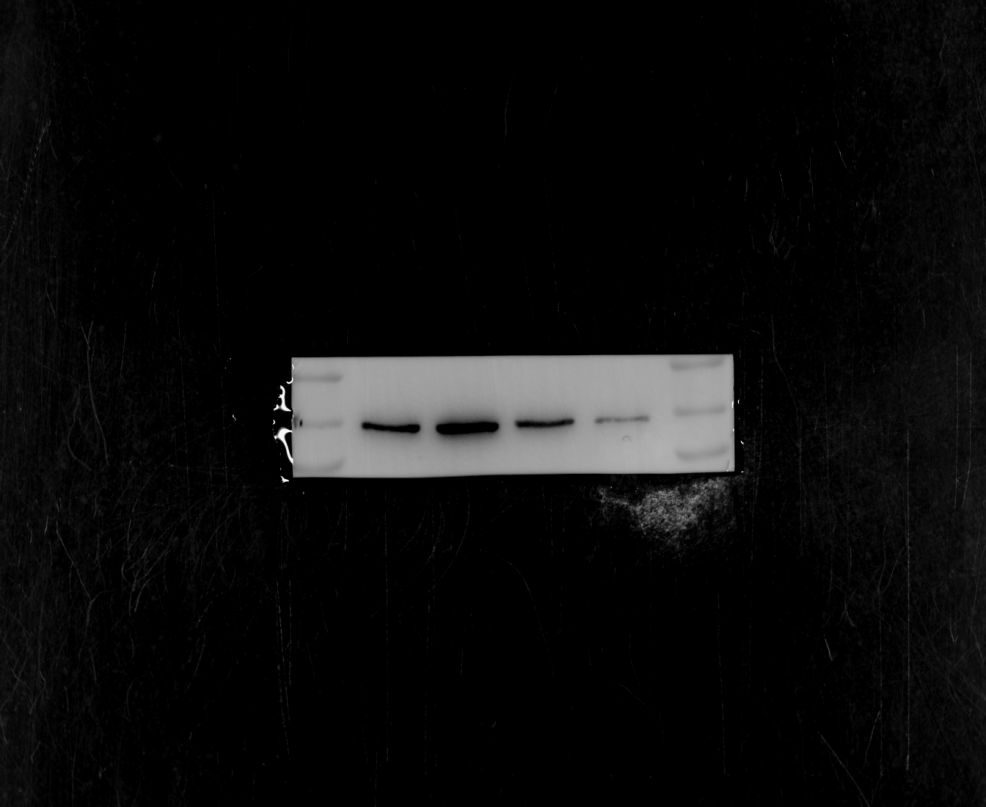
**

**β-actin**

**
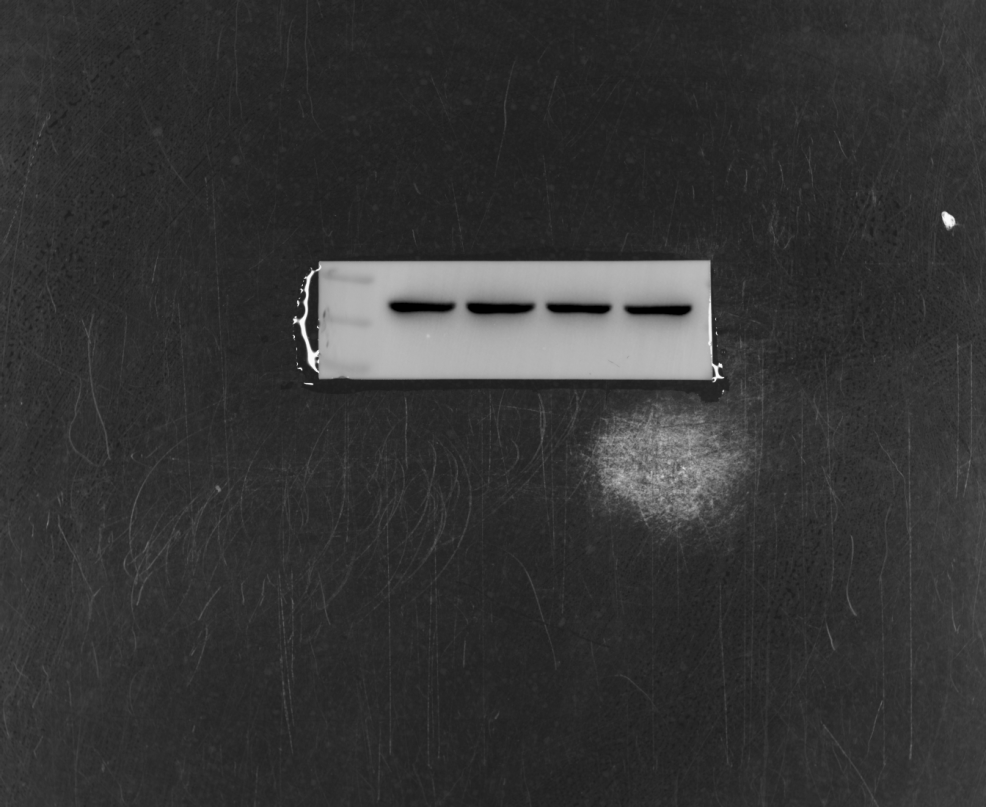
**

**Figure 6E**

**T24**

**
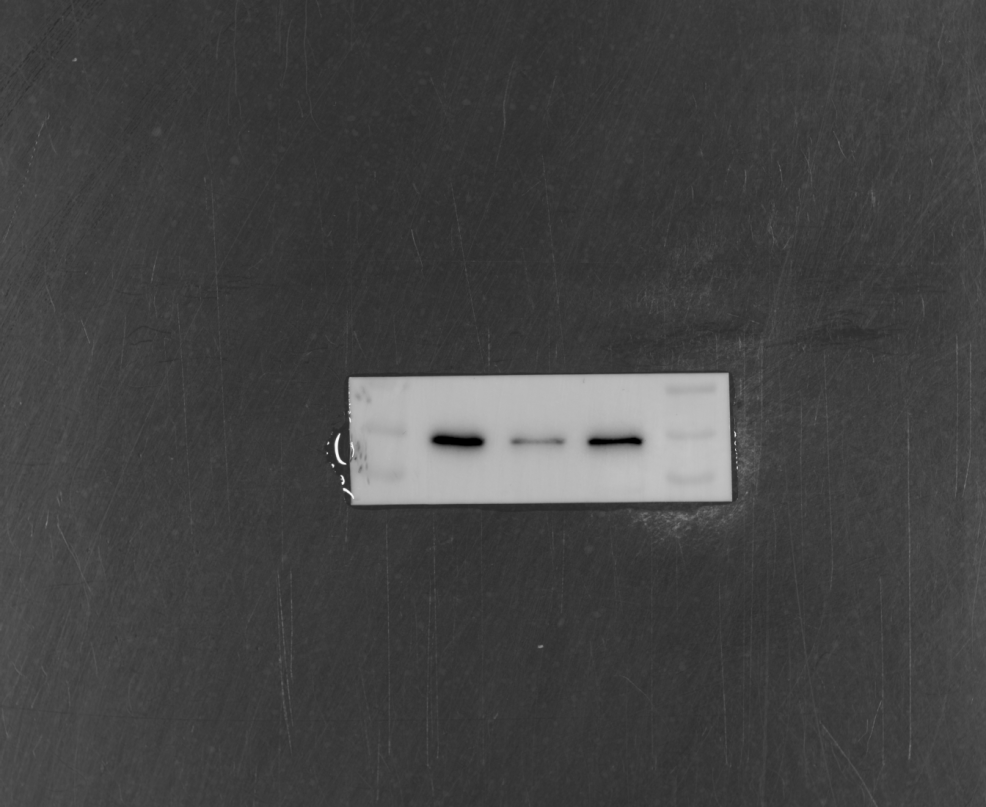
**

**β-actin**

**
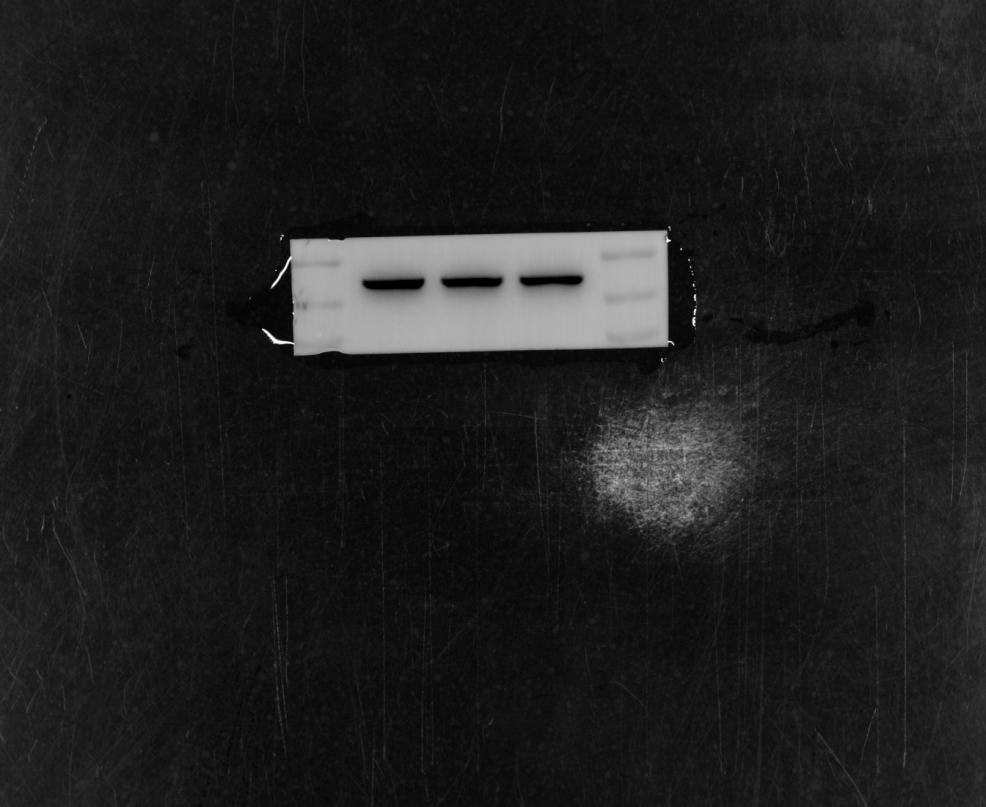
**

**5637**

**TRIM44**

**
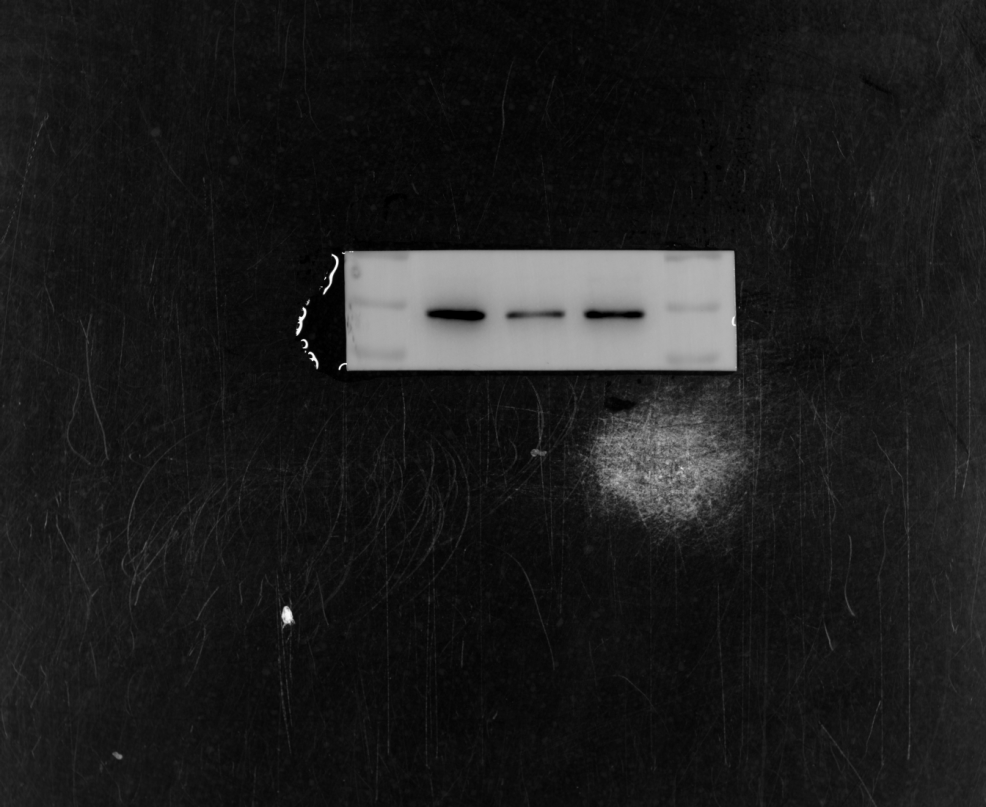
**

**β-actin**

**
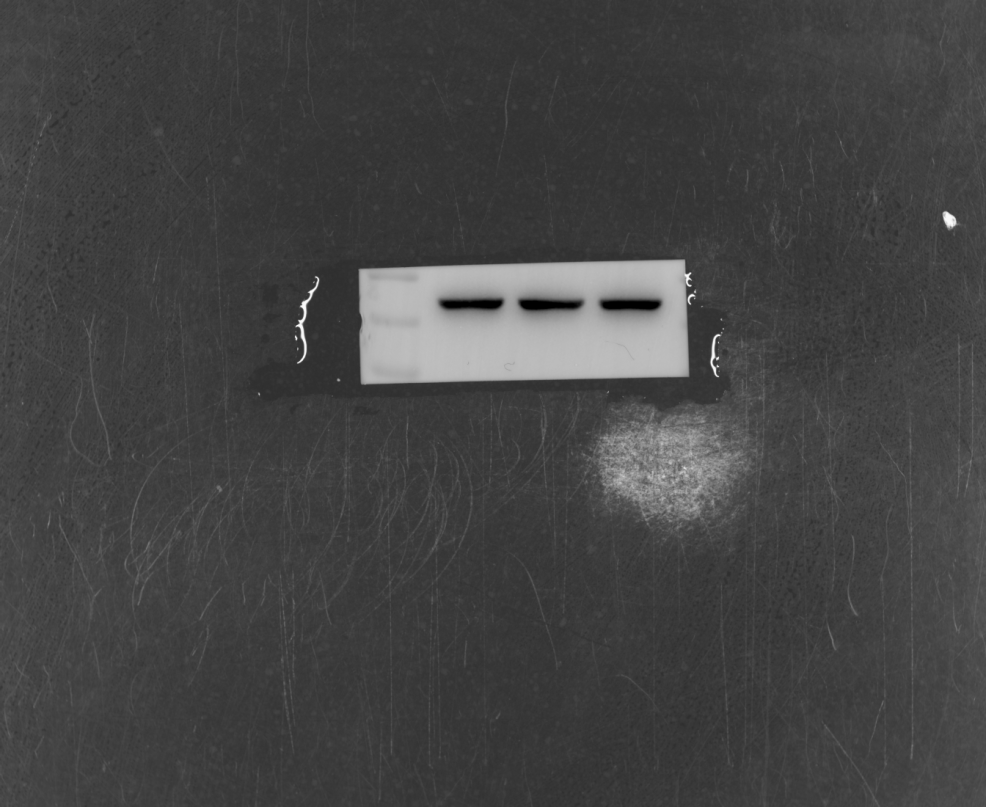
**

**Figure 7A**

**T24**

**TRIM44**

**
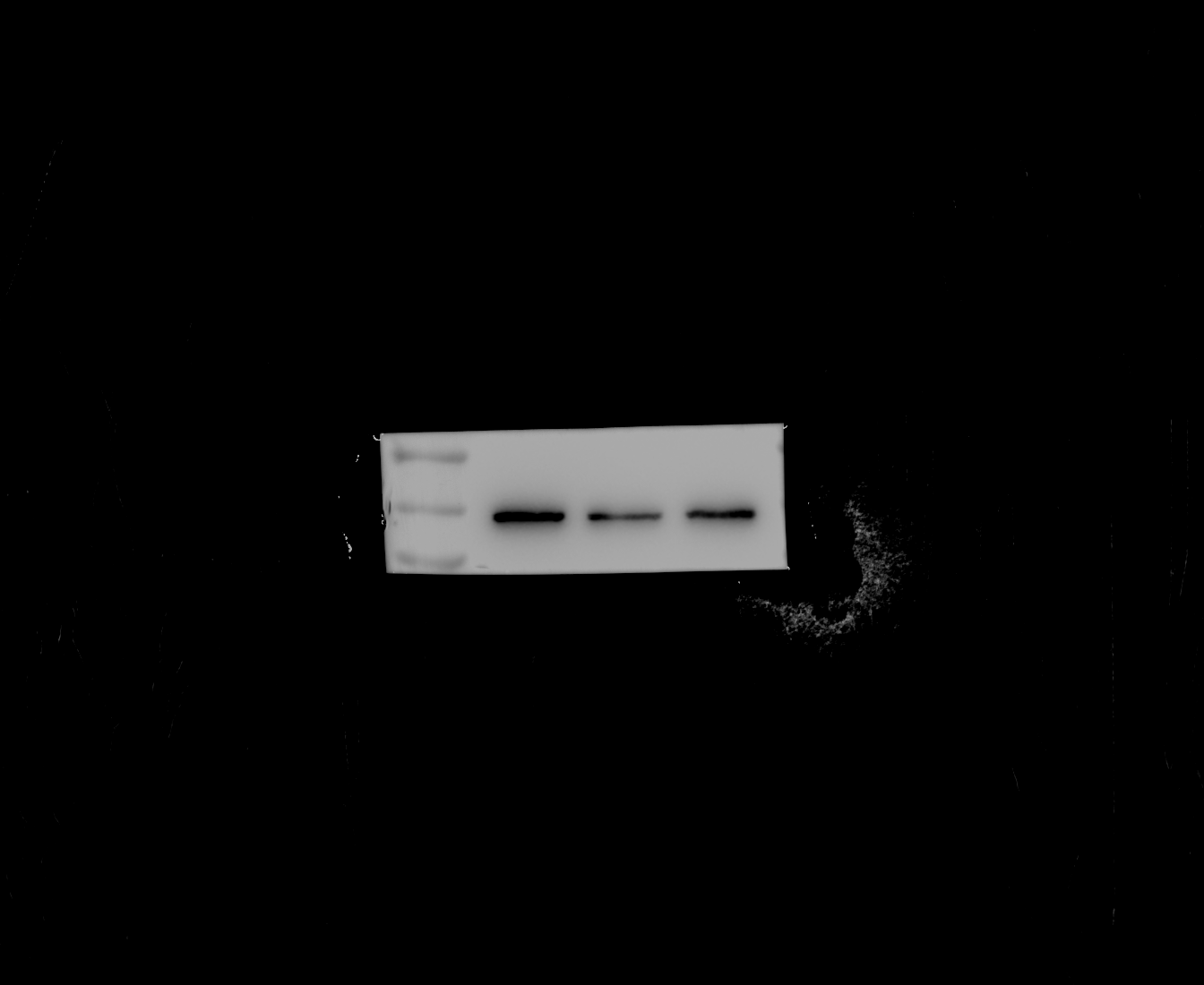
**

**β-actin**

**
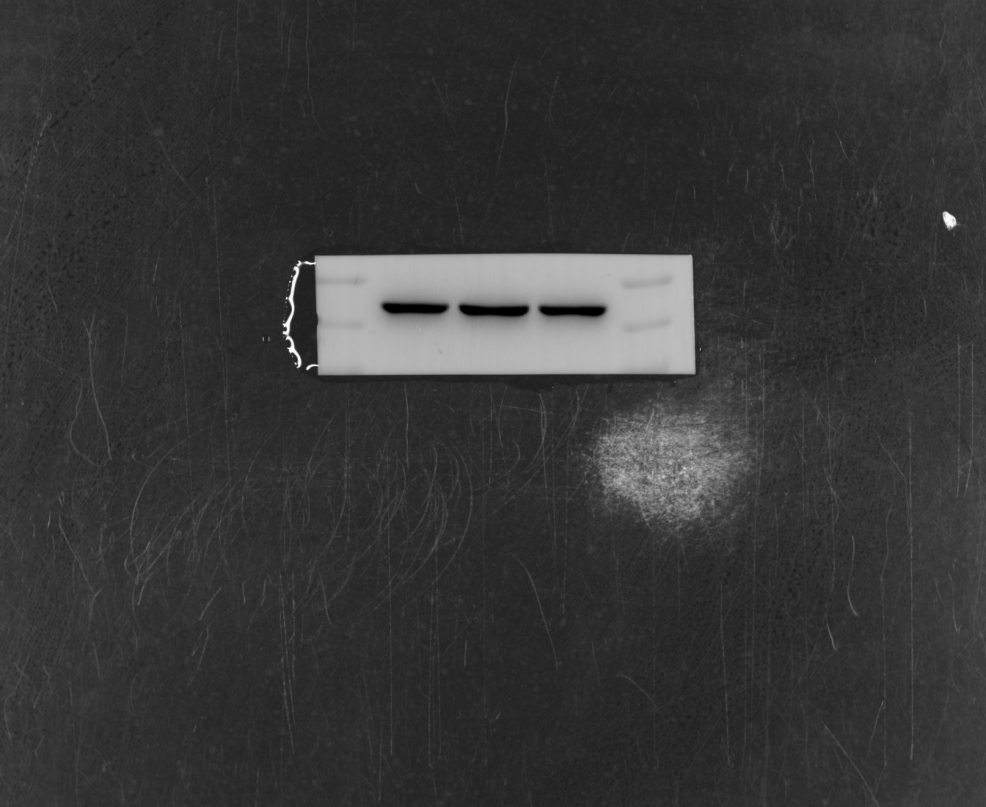
**

**5637**

**TRIM44**

**
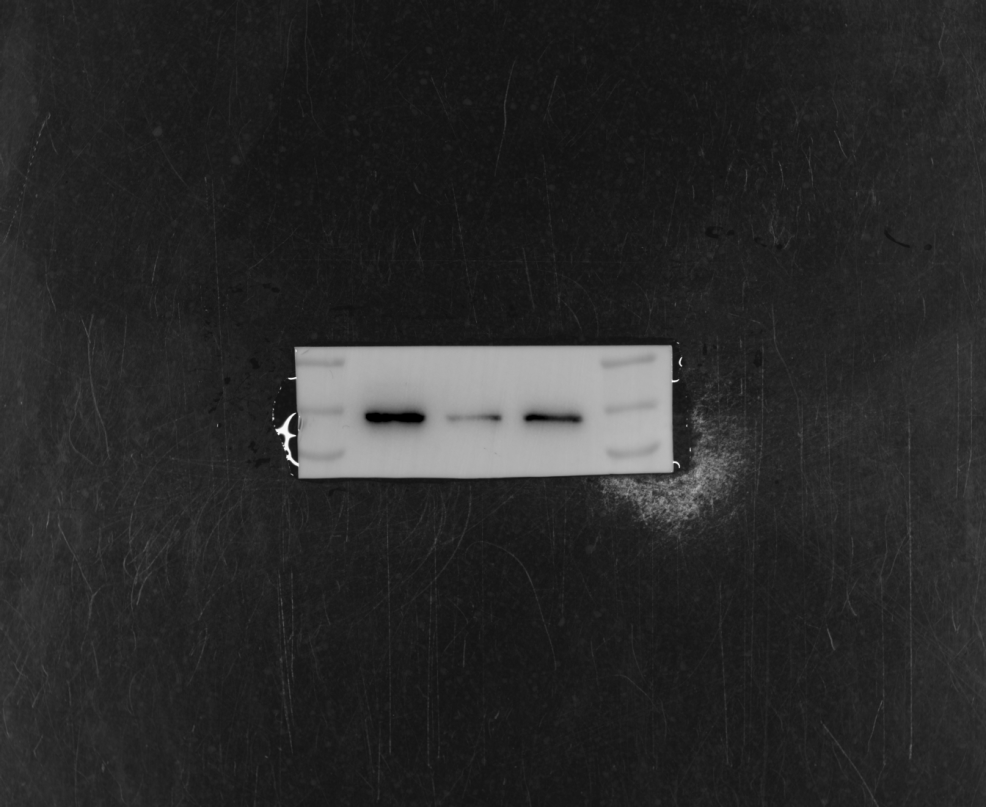
**

**β-actin**

**
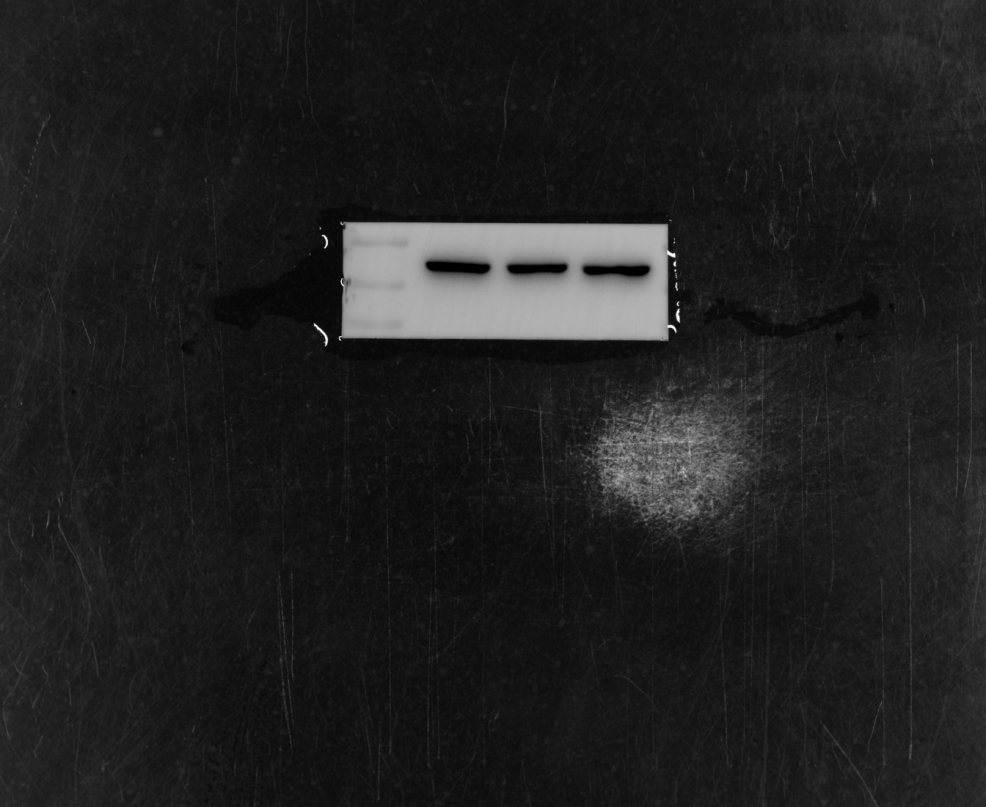
**

**Figure S1G**

**TRIM44**

**
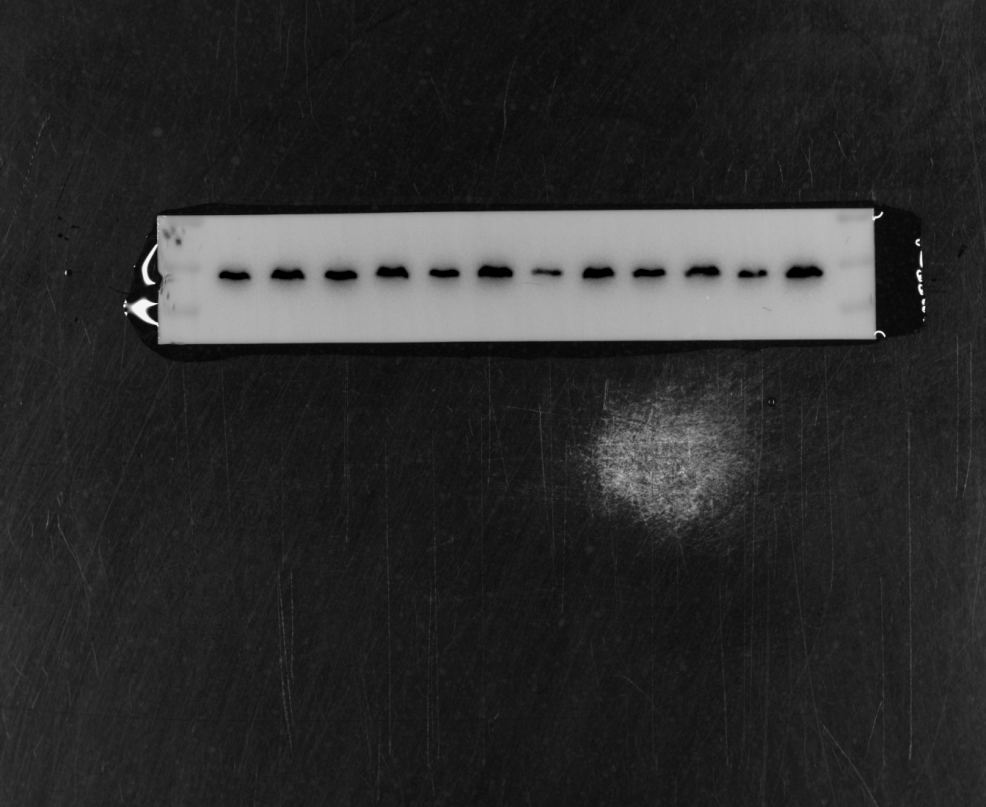
**

**β-actin**

**
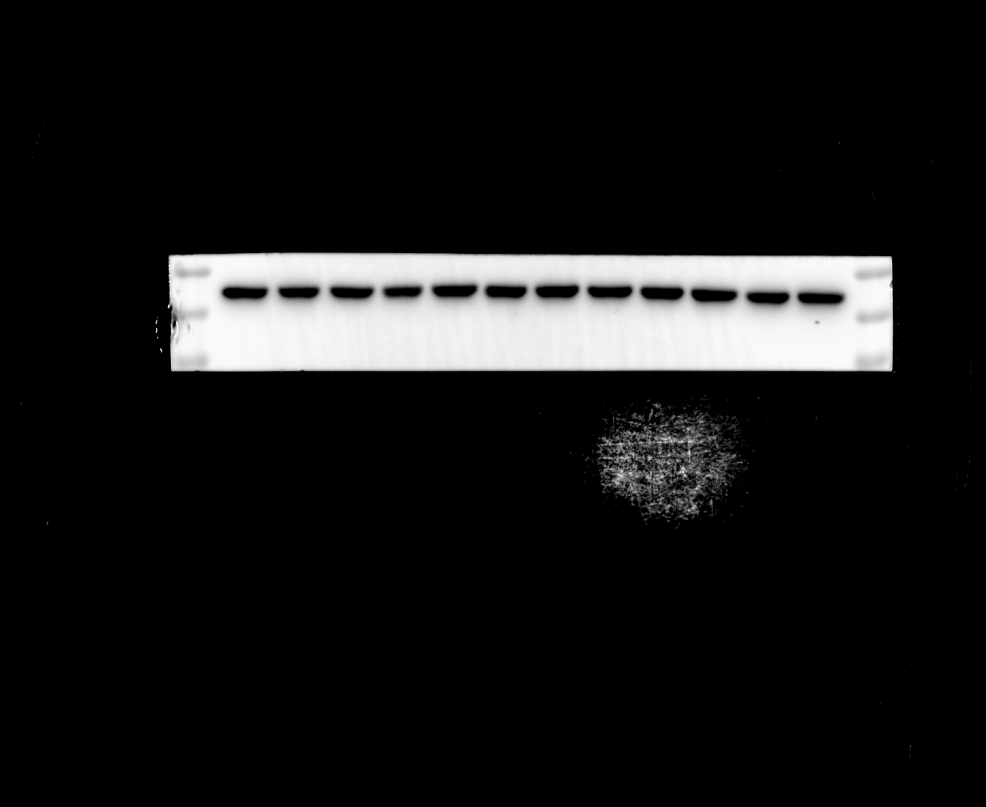
**
